# Process optimization of modified sodium persulfate for the remediation of total petroleum hydrocarbon contaminated soil

**DOI:** 10.1371/journal.pone.0331869

**Published:** 2025-09-12

**Authors:** Yuanyuan Li, Jinqiang Yang, Mingli Wei, Yaru Song

**Affiliations:** 1 School of Civil Engineering, Qingdao University of Technology, Qingdao, Shandong, China; 2 Jiangsu Institute of Zoneco Co., Ltd., Yixing, Jiangsu, China; 3 State Key Laboratory of Geomechanics and Geotechnical Engineering, Institute of Rock and Soil Mechanics, Chinese Academy of Sciences, Wuhan, Hubei, China; University of Greenwich, UNITED KINGDOM OF GREAT BRITAIN AND NORTHERN IRELAND

## Abstract

Petroleum hydrocarbon (TPH) contamination at industrial sites poses severe ecological and health risks to humans. However, conventional persulfate oxidation suffers from low efficiency and high oxidant demand requirements. To address this limitation, we employed citric acid-FeSO₄-modified sodium persulfate (FNS) for soil remediation.In this study, the petroleum hydrocarbon (TPH)-contaminated soil of a chemically contaminated site was used as the test object, and sodium persulfate modified by citric acid-FeSO_4_ (FNS) was selected as the oxidant. Based on laboratory tests, quantifying TPH degradation efficiency, soil pH variation, and sulfate leaching concentrations, the effects of oxidant dosage, oxidant dosage times, and CaO dosage on the remediation effect of petroleum hydrocarbon-contaminated soil were systematically investigated. The results demonstrated that citric acicd-FeSO_4_ significantly enhanced sodium persulfate activation (p < 0.01), achieving within 28 days a 36% higher TPH degradation efficiency than unmodified persulfate at 2% dosage. This modification amplified oxidation intensity and efficiency by up to 2.3-fold over the remediation period.For the contaminated soil with a petroleum hydrocarbon content of 16524 mg/kg, after a 28-day remediation period,FNS amendment achieved significant TPH reductions of 76.9% (to 3820 mg/kg) at 4% dosage-below Class II construction land in China’s soil environmental quality standard (4500 mg/kg). At a 6% dosage, after the same 28-day remediation period,reduction efficiency reached 95.5% (to 750 mg/kg) lower than the screening value of soil contamination risk for Class I construction land in China’s Soil Environmental Quality Standard (826 mg/kg). The FNS agent can significantly improve the oxidation strength and efficiency of sodium persulfate, but it causes soil acidification and exceeds the SO_4_^2-^ leaching concentration standard, among other things. The restored soil needs to be conditioned with CaO neutralization. In addition, FNS must be applied at one time and cannot be applied separately.

## Introduction

Total petroleum hydrocarbon(TPH) is a type of toxic pollutant commonly found in industrial polluted sites in China, and petroleum hydrocarbon pollution seriously threatens the surrounding ecological environment and people’s health [1,2]. Chemical oxidation is the most commonly used remediation technology for petroleum hydrocarbon-contaminated soil, which is characterized by its rapid effect, low cost, and wide application [3]. Sodium persulfate (Na_2_S_2_O_8_) is one of the most commonly used strong oxidizing agents and has been widely used in chemical oxidation technologies. Numerous engineering experiences have shown that although sodium persulfate has a good remediation effect on petroleum-hydrocarbon-contaminated soil, it has the disadvantages of large additive quantity, low remediation efficiency, and high cost. Improving the oxidizing strength and efficiency of sodium persulfate is an urgent engineering challenge [4,5]. Chemical activation is an effective method for improving the oxidation strength and efficiency of persulfate. Some scholars have conducted related research involving activation methods, including thermal, alkali, and metal ion activation. However, most of the above activation methods have the disadvantages of high energy consumption, many processes, and poor practicality [6,7].

**Fig 1:**
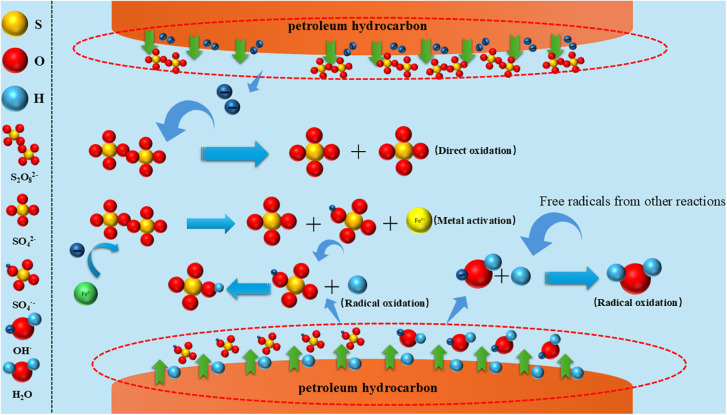
Remediation mechanism of sodium persulfate.

In this study, a modified sodium persulfate composite (FNS) was developed. The material was modified with citric acid-FeSO_4_ to strengthen the oxidizing and long-lasting properties of sodium persulfate, and its main repair mechanism is illustrated in Fig 1. In this study, cohesive contaminated soil from a typical chemical contaminated site in central China was selected as the test object.Based on indoor experiments, the effects of oxidant dosage, oxidant dosing times, and calcium oxide dosage on the remediation of petroleum-hydrocarbon-contaminated soils were systematically investigated under a remediation period of 28 days. The results of this study can provide key parameter support and technical guidance for the design, cost estimation, and remediation of chemical oxidation programs for clayey organic contaminated soils in central China.

Building on current limitations, we hypothesize that:Firstly,the synergistic activation of citrate-Fe² ⁺ overcame the slow generation of free radicals in the conventional persulfate system, and thus the degradation of petroleum hydrocarbons by modified sodium persulfate was higher than that of Na_2_S_2_O_8_;Secondly,a single application of modified sodium persulfate (FNS) is essential to maintain the free radical flux, and it can achieve a higher degradation efficiency than the batch application at a fixed total amount of application, which is particularly suitable for the remediation of hydrocarbons in highly clayey granular soils; Finally,CaO conditioning can neutralize sulfate byproducts without compromising oxidation efficacy, achieving dual compliance with soil (GB36600) and leachate standards (GB 3838).

## 1 Materials and methods

### 1.1 Contaminated soil

**Fig 2:**
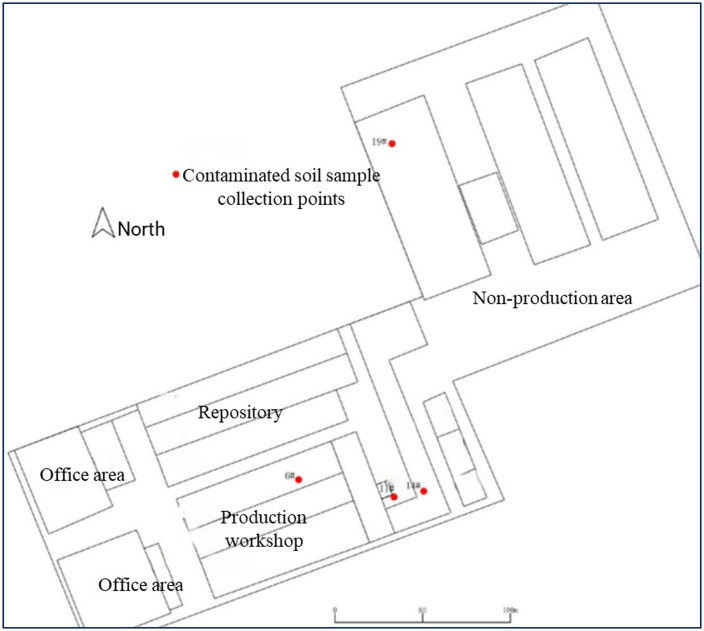
A schematic representation of the sampling locations at the contaminated site.

The study site (25°41′N, 119°20′E) is located in the Rongqiao Economic Development Zone,FuZhou City, FuJian Province.(Fig 2a), which is a topography that combines coastal plains and low-hill plateaus. The area was once the site of a chemical factory that mainly produced polyvinyl chloride (PVC), chlorinated benzene, and other chemical products, and serious petroleum hydrocarbon pollution was left after the relocation of the enterprise. The stratigraphic structure of the site is as follows: 0.6-1.9 m: miscellaneous fill (mixed with construction waste and pulverized soil); 3–12 m: pulverized clay layer; > 12 m: sand and gravel pressurized aquifer. The water table is buried at a depth of 3–5 m and is affected by seasonal fluctuations in the water level of the Yangtze River. Pollution investigation showed that the concentration of TPH was enriched at a depth of 8–10 m, with a concentration of 16524 mg/kg. This layer is located at the top of the capillary zone, where the water-holding capacity of the clay leads to pollutant retention. Sampling points were located along the historical production plants (Fig 2b).

The contaminated soil used in the test was collected from a chemical waste site in the Rongqiao Economic Development Zone, FuZhou City, FuJian Province, which mainly produces caustic soda, polyvinyl chloride, chlorinated benzene, lecithin, carbon black, and other chemical products, and has now been relocated and closed. According to the site investigation data and site conditions, the sampling depth of the contaminated soil was approximately 8–10 m below the ground. The contaminated soil belonged to powdery clay, and the characteristic pollutant of this site was mainly petroleum hydrocarbon, with a content of 16524 mg/kg. The characteristic parameters of the contaminated soil are presented in Table 1. The representative contaminated soil was first selected to remove stones and other impurities, and the samples were quickly sealed in self-sealing bags and stored under light-avoidance and low-temperature conditions.

**Table 1:** Physical, chemical indexes and pollutant content of contaminated soil.

Contaminated Soil Indicator	Parameter value
Natural moisture contentω_n_(%)	37.6
Plastic limitω_p_(%)	16.5
Liquid limitω_L_(%)	28.6
ProportionGs	2.67
Sticky grain content(%)	33.5
Powder content(%)	48.6
Sand content(%)	17.9

### 1.2 Test methods

First, the contaminated soil collected from the site was crushed and sieved to remove stones and other impurities. Then, FNS agent was added to the contaminated soil, tap water was used to adjust the water content of the contaminated soil, FNS agent to 30%, mixing well with transferring to the beaker, and finally sealing the beaker with plastic wrap and maintaining it at room temperature for 28 d. In addition, a portion of the contaminated soil taken from the site was retained as a field control (C0).The test program is presented in Table 2 (The percentage of agent additions in this study was based on the total soil mass(w/w).). In the design of the dosing program, when the split-dosing strategy was adopted, the specific implementation parameters were as follows: for the double-dosing condition, the FNS restoration chemicals were accurately weighed according to 4% of the total mass of the soil samples, and two groups of specimens were formed after equal equalizing, and the dosing operation was carried out in the beginning (0d) and middle stage (14d) of the maintenance cycle, respectively; when the triple-dosing program was adopted, the dosing was evenly divided into three equal portions, and the dosing operation was completed in the initial stage (0d) and, early stage (7d) and middle stage (14d). When the three-dose program was used, the quantitative agent was divided into three equal parts, and the dosing operation was completed at the initial (0d), early (7d) and intermediate (14d) phases of the maintenance cycle. The standard maintenance cycle of 28 d was maintained for all groups, and samples were collected and analyzed at the end of the maintenance cycle (28 d).

**Table 2:** Test design details.

Serial	Seria number	Na_2_S_2_O_8_ dosage (%)	FNS dosage (%)	Number of times added	CaO dosage(‰)
1	C0	0	0	0	0
2	N1	2	0	1	0
3	F1	0	2	1	0
4	F2	0	4	1	0
5	F3	0	6	1	0
6	F4	0	8	1	0
7	A1	0	4	1	0
8	A2	0	4	2	0
9	A3	0	4	3	0
10	C1	0	8	1	0
11	C2	0	8	1	2
12	C3	0	8	1	4
13	C4	0	8	1	6
14	C5	0	8	1	8

**Fig 3:**
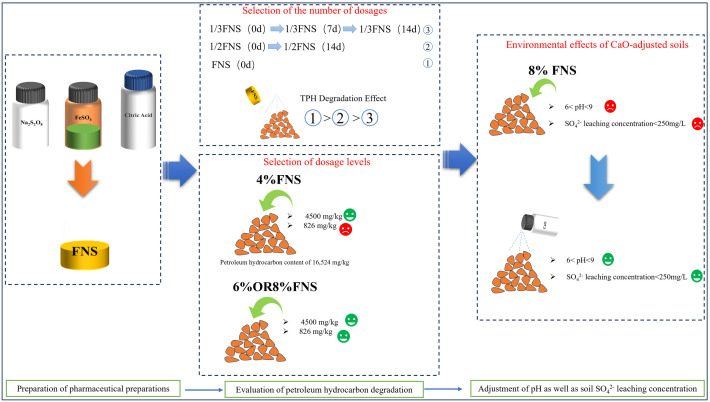
Process flow diagram for remediation of contaminated soil with modified sodium persulfate.

Each pollutant was tested according to the method given in the *Soil environmental quality Risk control standard for soil contamination of development land* (GB36600−2018 Chinese standard). The content of petroleum hydrocarbons in the soil was analysed using gas chromatography for petroleum hydrocarbon-contaminated soil [8]. Soil pH measurements were performed using a portable pH meter with a soil-water ratio of 1:2.5.The pH meter was calibrated using standard buffers of pH 4.0, 7.0, and 9.0 [9]. Determination of sulfate leaching concentration: A total of 150 g of sieved soil was mixed with deionized water leaching agent (liquid-solid ratio of 10:1) in a flip shaker for 18 h. The filtrate was filtered through a 0.45 μm filter membrane and analysed using ion chromatography [10].Three parallel samples were taken from each sample after the completion of the test, and the test results were averaged. After the test was completed, three parallel samples were taken for each sample, and the test results were averaged. The process flow diagram for this experiment is shown in Fig 3.

### 1.3 Quality assurance and control (QA/QC)

All analytical procedures followed China’s national standards with rigorous QA/QC protocols:

① Sample collection:

Field blanks (deionized water) transported with samples (1 per 10 samples).

GPS-documented sampling points (±3 m accuracy).

② Pre-treatment:

Matrix spike recovery: 92−107% for TPH (GB 36600−2018).

Duplicate analysis: RSD < 5% (n=3 per batch).

③ Instrument calibration: Refer to Table 3
④ Data validation:

**Table 3:** Instrument calibration parameters.

Analyte	Method	LOD	LOQ	Calibration R²
TPH	GC-FID	1 mg/kg	5 mg/kg	≥0.999
SO₄²	IC	0.1 mg/L	0.5 mg/L	≥0.999

Continuing calibration verification (CCV) every 10 samples.

Control charts for reference materials (NIST SRM 2711a).

## 2. Results and discussion

### 2.1 Soil pH

**Fig 4:**
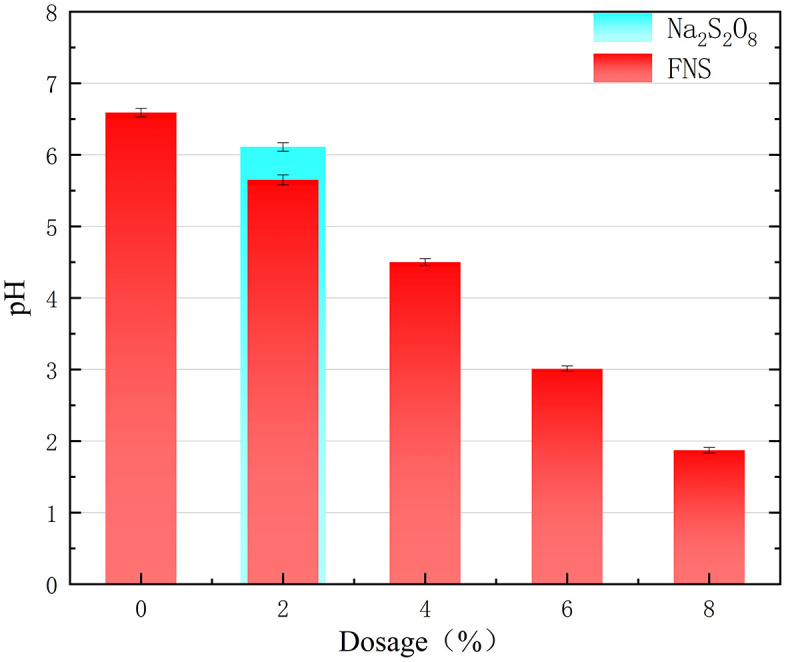
Effect of different FNS and Na_2_S_2_O_8_ additions on soil pH values.


The influence of remediation agent dosing on the pH of the contaminated soil is shown in Fig 4. As shown in Fig 4, the original pH of the polluted soil was 6.81, which is nearly neutral. The addition of Na_2_S_2_O_8_ and FNS caused the pH of the soil to exhibit an acidification trend. Specifically, the addition of Na_2_S_2_O_8_ decreased the soil pH from 6.81 to 6.14 when the Na_2_S_2_O_8_ dosage was increased from 0% to 2%.The decrease in soil pH is that Na_2_S_2_O_8_ produces H^+^ in an aqueous environment, which reacts as shown in Equation. (1), resulting in a decrease in soil pH [11].

$$S_{2}O_{8}^{2-}+H_{2}O\to 2SO_{4}^{2-}+2H^{+}+\frac{1}{2}O_{2}$$ (1)

The pH decreased from 6.59 to 1.87 when the FNS dosing was increased from 2% to 8%. Soil pH decreased with an increase in FNS dosing. The reason for the decrease in soil pH is that FNS contains a citric acid and FeSO_4_ mixture, which leads to acidification of the soil. According to the requirements for secondary soil use, the pH of the remediated soil needs to meet the requirement of being located at 6–9 before it can be used for secondary use [12]. Therefore, the soil after remediation with the FNS agent needs to be neutrally conditioned before it can be used for secondary utilization.

**Fig 5:**
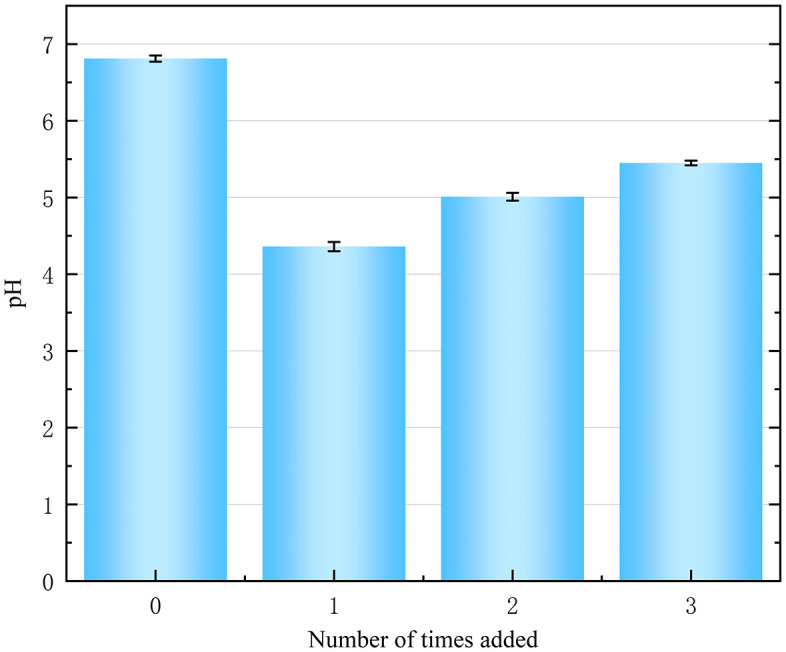
Effect of the number of FNS additions on soil pH.

The influence of the number of FNS additions on the pH of the contaminated soil is shown in Fig 5. As shown in Fig 5, the original pH value of the polluted soil was 6.81, which was nearly neutral. When the FNS dosage was 4%, the one-time and split FNS dosages had different effects on the soil pH. Specifically, the pH of contaminated soil tended to increase with the number of additions. When the number of additions increased from 1 to 3, the pH of the contaminated soil increased from 4.36 to 5.45 When we divided a certain amount of FNS remediation chemicals into multiple additions to contaminated soil (keeping the same amount each time), their ability to acidify the soil was weakened because the concentration of the chemical was diluted in each dose; therefore, as the number of times the agent was added increased, the cumulative effect of acidification of the soil by the agent weakened, ultimately leading to an increase in soil pH with the number of times the agent was added.

**Fig 6:**
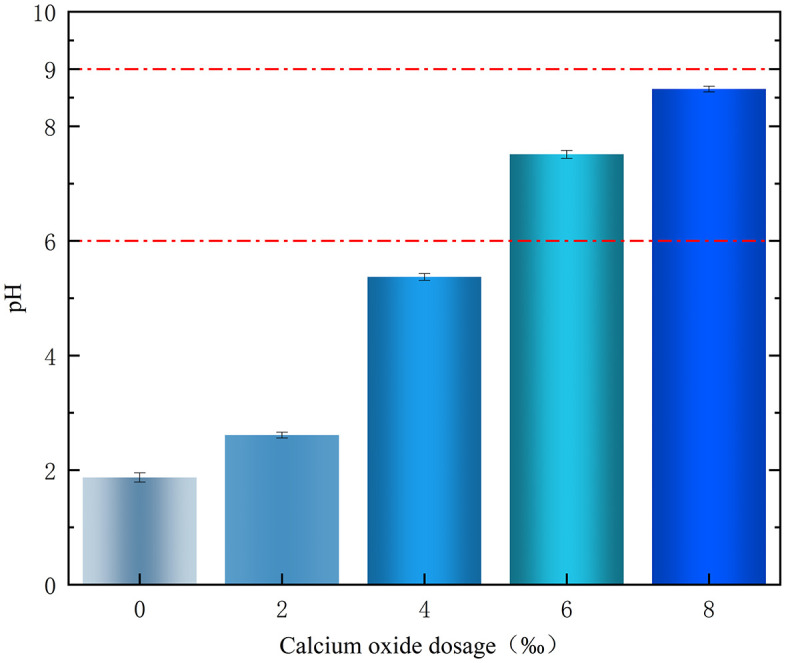
Effect of calcium oxide dosing on pH.

The effect of different CaO doping on the pH of contaminated soil is shown in Fig 6. As shown in Fig 6, when the FNS doping was 8%, the pH of the polluted soil increased continuously with an increase in CaO doping. When CaO doping increased from 0‰ to 8‰, the pH of the polluted soil increased from 1.87 to 8.65.

When the CaO doping was 6‰, the pH value of the polluted soil was 7.45, which met the pH range of heavy metal-polluted site soil as required by the *Standards for Soil Remediation of Heavy Metal Contaminated Sites* (DB43/T 1165–2016 Chinese standard). The increase in soil pH was attributed to the reaction of CaO with water to produce OH^-^. Therefore, the soil after remediation with the FNS agent can be neutrally adjusted by referring to the CaO dosage in Fig 6.

### 2.2 Soil petroleum hydrocarbon content

**Fig 7:**
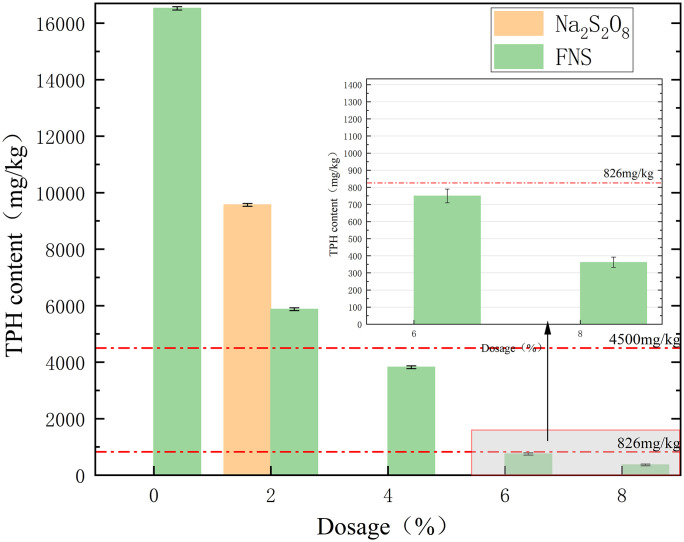
Effect of different FNS and Na_2_S_2_O_8_ additions on TPH content.

The influence patterns of different remediation chemicals on the soil total petroleum hydrocarbon (TPH) content are shown in Fig 7. The original TPH content of the contaminated soil was 16524 mg/kg, which far exceeded the risk screening value (4500 mg/kg) of soil contamination of Class II construction land in the Chinese soil environmental quality standard and had a large environmental risk. Na_2_S_2_O_8_ produces a large amount ofSO_4_^•-^under the effect of activation, which can rapidly react with petroleum hydrocarbon pollutants in the soil, thus reducing the TPH content. As shown in Fig 7, the addition of both Na_2_S_2_O_8_ and FNS significantly reduced the TPH content in the soil. Field control (C0) maintained initial TPH at 16524 ± 320 mg/kg. After 28 days of post-conservation parallel testing (n = 3), the rate of change in TPH concentration in the C0 group was less than 1.8%, indicating that the natural attenuation effect was negligible. The degradation results of each restoration group after 28 d of restoration were as follows:When the Na_2_S_2_O_8_ dosage was increased to 2%, the TPH content decreased to 9567 mg/kg, representing a 42.1% degradation rate relative to field control (C0: 16524 ± 320 mg/kg). When the FNS dosage was increased from 2% to 8%, the TPH content decreased from 5872 mg/kg(64.5% degradation vs.C0) to 362 mg/kg(97.8% degradation vs.C0). When the FNS dosage was 4%, the TPH content decreased to 3820 mg/kg(76.9% degradation vs.C0), the TPH content was lower than the screening value of the risk of soil contamination of construction land in Class II of the Chinese Soil Environmental Quality Standard (4500 mg/kg). When the FNS content was 6%, the TPH content decreased to 750 mg/kg(95.5% degradation vs.C0),the TPH content was lower than the screening value of soil contamination risk for Class I construction land (826 mg/kg) in the Chinese soil environmental quality standard. Analyzing the test data in Fig 7, it can be seen that the TPH content of Na_2_S_2_O_8_ remediated soil is higher than that of FNS remediated soil at the same dosage. This is due to the obvious difference in the intensity of the oxidation reaction between the two. In acidic solution, the oxygen-oxygen bond of Na_2_S_2_O_8_ breaks asymmetrically to form sulfate radicals and bisulfate.The activation energy of the acid-catalyzed reaction of Na_2_S_2_O_8_ is 108.8 kJ/mol and is smaller than that of the non-catalyzed reaction of Na_2_S_2_O_8_ (140 kJ/mol) [13,14].The FNS agent acidifies Na_2_S_2_O_8_, which improves the oxidation reaction capacity and increases the TPH degradation efficiency. This proves that the modified Na_2_S_2_O_8_ is more effective in degrading petroleum hydrocarbons [15–17].

**Fig 8:**
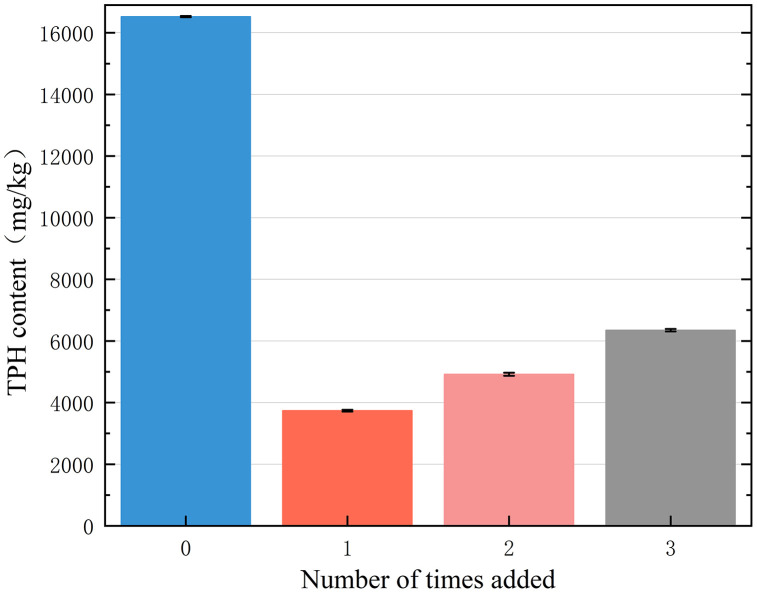
Effect of the number of FNS additions on soil TPH content.


The effects of different remediation chemical addition times on the TPH content of the contaminated soil are shown in Fig 8. When the FNS dose was 4%, the one-time and sub-dosing of FNS had different effects on the TPH content of the soil. The TPH content showed an increasing trend with an increase in the number of additions. Against the initial TPH (16524 mg/kg), a single FNS application (4%) reduced contamination to 3820 mg/kg(76.9% degradation vs.C0), whereas split dosing yielded only 6349 mg/kg(61.6% degradation vs.C0) after three additions. The experimental results showed that adding the same total amount of FNS to the soil in several times, on the contrary, reduced the remediation effect. TPH removal was higher when added in a single addition, whereas split addition resulted in higher pollutant residues. This is because split addition results in an insufficient concentration of free radicals (SO_4_^•-^), which weakens the degradation of petroleum hydrocarbons [18–20]. In addition, the pH of the contaminated soil increased after the batch addition of the FNS. As pH increased, the OH-content increased, and Fe(II) was easily oxidized to Fe(III) and precipitated together with OH^-^, as shown in Equation. (2), (3), and (4), leading to a decrease in degradation efficiency [11]. Therefore, an acidic pH is more favorable for the degradation of petroleum hydrocarbons than neutral and basic pH [21].

$${Fe}^{\text{2}+}\to {Fe}^{\text{3}+}+e^{-}$$ (2)

$${Fe}^{\text{2}+}+\text{2}OH^{-}\to Fe{\left(OH\right)}_{\text{2}}$$ (3)

$${Fe}^{\text{3}+}+\text{3}OH^{-}\to Fe{\left(OH\right)}_{\text{3}}$$ (4)

**Fig 9:**
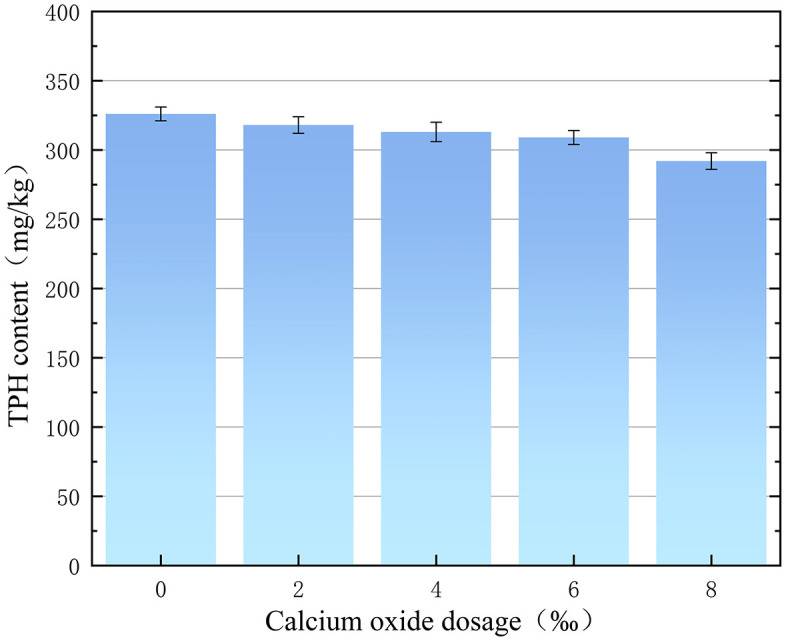
Effect of calcium oxide dosing on TPH content.

The effect of different CaO doping on the TPH content of the contaminated soil is shown in Fig 9. As shown in Fig 9, when the FNS doping is 8%, the TPH content of the contaminated soil shows a slight decreasing trend with the increase of CaO doping.When the amount of CaO was increased from 0‰ to 8‰, the TPH content of the contaminated soil was reduced from 326 mg/kg to 292 mg/kg, realising an additional 10.4% reduction.

The reason for the decrease in the TPH content of the contaminated soil is that CaO reduces the water content of the contaminated soil, increases the dispersibility and porosity of the soil, and releases the TPH inside the soil particles to the surface, which facilitates the reaction between the residual FNS agent and TPH. Therefore, the TPH content of polluted soil showed a slight decreasing trend with the increase of CaO doping [22–24].

Although the additional TPH reduction from CaO conditioning was only 10.4%, its engineering value was significant. In terms of risk control, CaO reduced the TPH from 326 mg/kg (39.5% near the Class I standard limit of 826 mg/kg) to 292 mg/kg (35.3%). In terms of cost-effectiveness, for every 1‰ CaO added, TPH was reduced by 1.3% more, whereas the cost of CaO was only 1/20 of that of FNS. In addition, the addition of CaO was necessary to solve the problems of acidification and simultaneous sulphate leaching at the same time.

### 2.3 Sulfate radical leaching concentration

**Fig 10:**
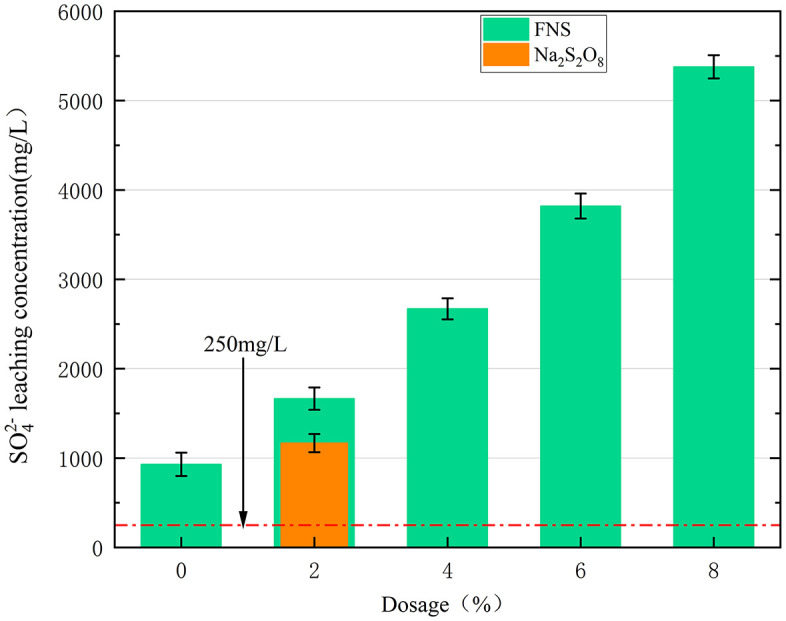
Effect of different FNS and Na_2_S_2_O_8_ additions on the leaching concentration of SO_4_^2-^.

The pattern of FNS and Na_2_S_2_O_8_ dosing on soil SO_4_^2-^ leaching concentration is shown in Fig 10. The background sulfate radical leaching concentration of the original contaminated soil (Field control C0) was 758 mg/L, indicating pre-existing sulfate contamination from the site’s history, which was higher than the centralised drinking water leaching standard (250 mg/L) in the environmental quality standard of China’s surface water (GB 3838−2002 Chinese standard). The addition of Na_2_S_2_O_8_ and FNS significantly increased the SO_4_^2-^ leaching concentration in soil. Specifically, when the Na_2_S_2_O_8_ dosage was increased to 2%, the leaching concentration of SO_4_^2-^ in the soil increased to 1167 mg/L. When the FNS dosage was increased from 2% to 8%, the leaching concentration of SO_4_^2-^ increased from 1665 mg/L to 5378 mg/L. The reason for the above results is that the FNS agent contains a large amount of Na_2_S_2_O_8_, which releases a large amount of SO_4_^2−^ as the reaction proceeds. A large amount of SO_4_^2-^ was released as the reaction proceeded, leading to an increase in the leaching concentration. Due to the high leaching concentration of SO_4_^2-^ in the remediated soil, it is necessary to neutralize and condition the soil after remediation with the FNS agent.

**Fig 11:**
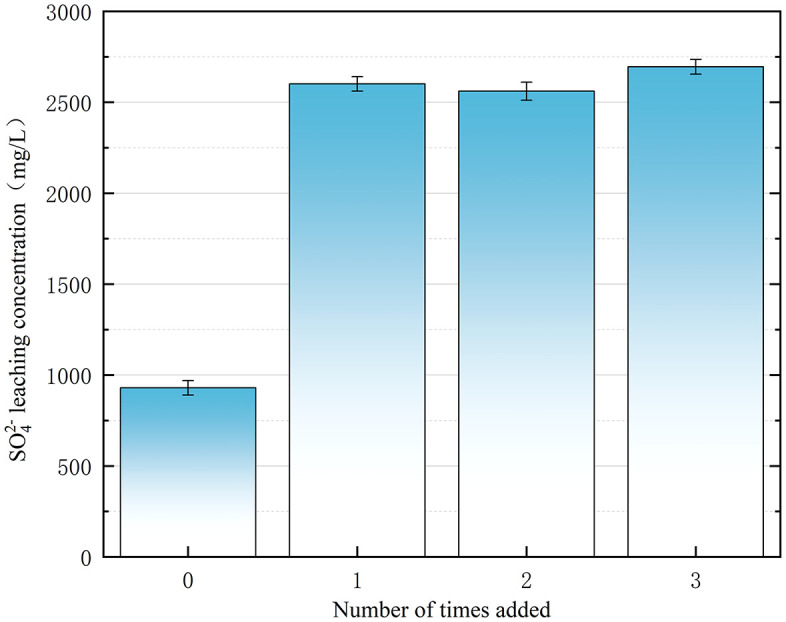
Effect of the number of FNS additions on the leaching concentration of soil SO_4_^2-^.

Fig 11 shows the variation in SO_4_^2-^ leaching concentrations with the frequency of FNS application (8% dosage). The background value before treatment was 758 mg/L (Section 2.3). When the frequency of FNS addition was increased from 1 to 3 times(2601 mg/L to 2695 mg/L), the SO_4_^2-^ leaching concentration of the contaminated soil varied between 2,561 mg/L (2 times) and 2,695 mg/L. Statistical analysis confirmed insignificant differences (p = 0.38 by t-test) in sulfate leaching between single and divided FNS dosing at 8% dosage (Fig 11). This is because sulfate liberation depends solely on the stoichiometric decomposition of persulfate (${Na}_{2}S_{2}O_{8}\to 2{Na}^{+}+2SO_{4}^{2-}$) and not on the frequency of application. The total oxidant mass determines the final sulfate yield, regardless of the delivery mode [24,25].

**Fig 12:**
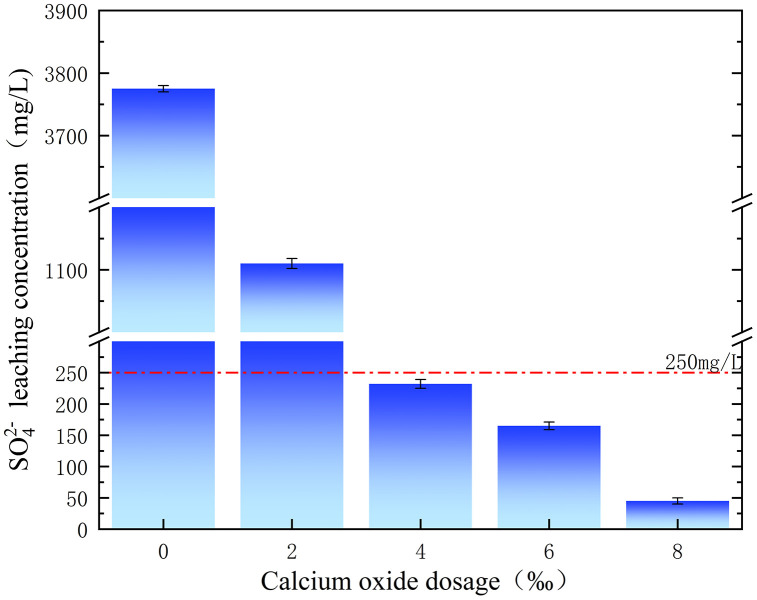
Effect of calcium oxide dosage on the leaching concentration of SO_4_^2-^.


The effects of different CaO dosages on the SO_4_^2-^ leaching concentration of polluted soil are shown in Fig 12. When the dosage of Na_2_S_2_O_8_ was 8%, the SO_4_^2-^ leaching concentration gradually decreased with increasing CaO dosage of calcium oxide. When the dosage of CaO increased from 0 to 8 ‰, the SO_4_^2-^leaching concentration of polluted soil decreased from 3775 to 45 mg/L. When the dosage of CaO was 4 ‰, the SO_4_^2-^leaching concentration was lower than that of the centralised drinking water leaching standard (250 mg/L) in China’s environmental quality standard for surface water (GB 3838−2002 Chinese standard). The reason for the above test results is the continuous decomposition of calcium oxide to form a large amount of Ca^2+^, which generates CaSO_4_ precipitation with SO_4_^2-^, leading to a continuous decrease in the SO_4_^2-^ leaching concentration in the soil [26,27], as shown in Equation. (5) and (6), respectively.

$$CaO+H_{2}O\to {Ca}^{2+}+2OH^{-}\;\;$$ (5)

$${Ca}^{2+}+{SO}_{4}^{2-}\to CaSO_{4}\;\;$$ (6)

## 3 Conclusion

In this study, cohesive contaminated soil from a typical chemical contaminated site in central China was selected as the test object, and citric acid-FeSO_4_-modified sodium persulfate (FNS) was chosen as the oxidising agent. Based on the batch indoor test, the influence of oxidant dosing, oxidant dosing times, and calcium oxide dosing on the remediation effect of petroleum-hydrocarbon-contaminated soil was systematically investigated. The research results can provide key parameter support and technical guidance for the design of chemical oxidation programs, cost estimation, and engineering remediation of clayey organic contaminated soils in central China. The following conclusions were drawn:

(1) Citric acid-FeSO_4_ had a good activation effect on sodium persulfate, and the oxidation strength and efficiency of the activated sodium persulfate were significantly improved. For soil contaminated with a petroleum hydrocarbon content of 16524 mg/kg, the TPH content was lower than the screening value of soil contamination risk for Class II construction land in China’s soil environmental quality standard (4500 mg/kg) when the FNS doping was 4%, and the TPH content was lower than the screening value of soil contamination risk for Class I construction land in China’s soil environmental quality standard (826 mg/kg) when the FNS doping was 6%.
(2) While FNS significantly improves remediation efficiency, its application causes significant soil acidification and elevated SO_4_^2-^ leaching. Therefore, post-oxidation neutralization and conditioning with calcium oxide are essential. Based on the observed drawbacks and remediation requirements:It is strongly recommended to apply calcium oxide immediately after FNS treatment. The optimal dosage ratio (CaO/FNS) should be determined based on site-specific soil conditions and the applied FNS dose to ensure effective pH neutralization (targeting near-neutral pH) and SO_4_^2-^ precipitation.FNS should be injected in a single application rather than separate doses to maximize oxidation efficiency and minimize potential negative impacts associated with multiple acidification events.
(3) Post-remediation monitoring is critical to verify long-term effectiveness and manage potential residual risks. A monitoring plan should include, at minimum:TPH concentrations in soil: To confirm ongoing compliance with the applicable risk screening values (Class I or II);Soil pH: To assess the stability of the neutralization effect and detect potential acidification rebound.SO_4_^2-^ concentrations in soi: To monitor leaching potential and prevent secondary groundwater contamination.Monitoring frequency should commence shortly after remediation completion (e.g., 1 week, 1 month) and continue periodically (e.g., quarterly, semi-annually) for at least one hydrological year or as required by regulations, based on site-specific risk assessment.

## Supporting information

S1 Data Dataset of soil properties and residual concentrations following remediation with modified sodium persulfate. (XLSX)
